# Simultaneous attenuation of trace organics and change in organic matter composition in the hyporheic zone of urban streams

**DOI:** 10.1038/s41598-021-83750-8

**Published:** 2021-02-18

**Authors:** Birgit M. Mueller, Hanna Schulz, Robert E. Danczak, Anke Putschew, Joerg Lewandowski

**Affiliations:** 1 Department Ecohydrology, Leibniz Institute of Freshwater Ecology and Inland Fisheries, Mueggelseedamm 310, 12587 Berlin, Germany; 2 Department of Environmental Sciences and Technology, Chair of Water Quality Engineering, Technische Universität Berlin, Straße des 17. Juni 135, 10623 Berlin, Germany; 3 Geography Department, Humboldt University of Berlin, Rudower Chausee 16, 12489 Berlin, Germany; 4 Earth and Biological Sciences Directorate, Pacific Northwest National Laboratory, 902 Battelle Blvd., Richland, WA 99352 USA

**Keywords:** Biogeochemistry, Environmental sciences, Hydrology, Limnology

## Abstract

Trace organic compounds (TrOCs) enter rivers with discharge of treated wastewater. These effluents can contain high loads of dissolved organic matter (DOM). In a 48 h field study, we investigated changes in molecular composition of seven DOM compound classes (FTICR-MS) and attenuation of 17 polar TrOCs in a small urban stream receiving treated wastewater. Correlations between TrOCs and DOM were used to identify simultaneous changes in surface water and the hyporheic zone. Changes in TrOC concentrations in surface water ranged between a decrease of 29.2% for methylbenzotriazole and an increase of 152.2% for the transformation product gabapentin-lactam. In the hyporheic zone, only decreasing TrOC concentrations were observed, ranging from 4.9% for primidone to 93.8% for venlafaxine . TrOC attenuation coincided with a decline of molecular diversity of easily biodegradable DOM compound classes while molecular diversity of poorly biodegradable DOM compound classes increased. This concurrence indicates similar or linked attenuation pathways for biodegradable DOM and TrOCs. Strong correlations between TrOCs and DOM compound classes as well as high attenuation of TrOCs primarily occurred in the hyporheic zone. This suggests high potential for DOM turnover and TrOC mitigation in rivers if hyporheic exchange is sufficient.

## Introduction

Urban streams are impacted by high loads of nutrients and contaminants^1^ such as trace organic compounds (TrOCs). The widespread occurrence of TrOCs threatens the quality of drinking water resources^2–4^ and alters freshwater ecosystems^5,6^.TrOCs enter urban waters, besides other pathways, especially with discharge of treated wastewater^7^. In addition to TrOCs, effluents of wastewater treatment plants (WWTP) contain elevated loads of organic matter^8^. Although a variety of laboratory studies found differing impacts of organic matter on the attenuation of TrOCs^9–12^, knowledge on potential simultaneous turnover of these compounds in a real river and its hyporheic zone is still lacking.

In aquatic systems, the main attenuation pathway for TrOCs is microbial biotransformation^13^. Due to steep physical and biogeochemical gradients, the hyporheic zone is a hotspot for microbial biodiversity and increased biogeochemical reaction rates^14^, including the attenuation of TrOCs^15^. Selected TrOCs are also affected by photolysis^16,17^ or sorption^18^. Other important factors influencing the attenuation of TrOCs are temperature^19,20^, redox conditions^21^, chemical characteristics of the substance itself^22^, and organic carbon including its bioavailability^23^.

The results of different studies on how dissolved organic carbon (DOC) influences the attenuation of TrOCs are contradictory. Foulquier et al.^24^ found a positive relationship between the concentration of bioavailable DOC (BDOC) and microbial biomass as well as activity in a combined field and column experiment. Similarly, TrOC biodegradation increased with higher BDOC concentrations in a batch test^9^, soil columns^10^ or a combined setup with acidic TrOCs^23^. These findings suggest that microbial co-metabolism with BDOC as the primary carbon source enables the attenuation of TrOCs^10^. However, it was also observed that the attenuation of 9 out of 14 TrOCs^25^ or hydrophilic neutral TrOCs^23^ was independent of the BDOC concentration.

In contrast, competition between BDOC and TrOCs was found in other soil column experiments^11,12^. These studies observed higher TrOC attenuation under lower BDOC concentrations. This relationship was linked to a more diverse microbial community under limited BDOC availability. A non-adapted microbiological community attenuated TrOCs better at higher BDOC concentrations while adapted microbes showed similar or better TrOC attenuation under low BDOC conditions in similar experiments^10,26^. In all studies mentioned, sorption was either not evaluated or negligible for most TrOCs.

The exact composition of carbon sources is well known in controlled laboratory studies. In previous experiments, the parameter defining primary substrate was usually B(DOC). In contrast, a huge variety of potential primary substrates from the complex pool of dissolved organic matter (DOM) compounds occurs in natural systems. Still, DOC is often used as a proxy for DOM as its analysis is straightforward. In contrast to DOC, DOM mostly consists of carbon, phosphorus, nitrogen, and oxygen. To account for a diverse organic matter composition in a river^27^, an extension of DOC to DOM is beneficial.

One of the various methods to measure DOM is Fourier transform ion cyclotron resonance mass spectrometer (FTICR-MS) analysis. Using FTICR-MS, molecular formulas are identified and can be matched with different DOM compound classes with varying bioavailability. While FTICR-MS analysis offers high resolution data, it only allows for a qualitative or semi-quantitative approach. Therefore, concentrations of different compounds remain unknown. Instead, the number of molecular formulas in a DOM compound class can be compared between samples^27^ to analyse changes in DOM composition.

DOM bioavailability not only depends on its composition^28^ but also on microbial activity linked to oxygen concentrations^29^ and nutrient availability^28^. Close hydrological connectivity between surface water and the hyporheic increases DOM bioavailability by supplying hyporheic microbes with oxygen and DOM itself^30^. In the same river system as our field site (River Erpe, East of Berlin, Germany), Schaper et al.^31^ observed attenuation of TrOCs occurring parallel to a decrease in DOC concentration and bioavailability in the hyporheic zone. Due to discharge of treated wastewater to the stream, elevated nutrient loads characterize this field site, indicating that DOM bioavailability is not nutrient limited^28^. Other studies at the River Erpe also found attenuation of TrOCs in the hyporheic zone^21,32^ as well as in surface water^33^ but did not compare their observations to changes in DOM.

Regarding the interaction between DOM composition and TrOC attenuation, field studies are severely underrepresented. While laboratory studies under controlled conditions help to focus on selected processes, simplification of the system is inevitable. However, simplification might also lead to the exclusion of important processes which would occur in a natural system. Therefore, the objective of the present field study is to investigate in situ the combined fate of TrOCs and DOM composition in both, surface water and the hyporheic zone of an urban stream.

The hyporheic zone and surface water of the side channel “Rechter Randgraben” are compared in both, changes in TrOC concentrations and DOM molecular diversity as well as in simultaneity of these changes. Our hypotheses are: (1) In urban streams with elevated nutrient loads, TrOC attenuation coincides with changes in DOM molecular diversity, especially for DOM compound classes with high bioavailability, along a surface water flow path and a flow path into the hyporheic zone. (2) Changes in TrOCs and DOM, as well as similarity in these changes, mainly occur in the hyporheic zone and not in surface water.

## Results

### Sediment characteristics, redox conditions and nutrient availability

Streambed sediment analysis at the pore water sampling site of the side channel “Rechter Randgraben” indicated a top sandy layer in 0 to 4–9 cm depth and an underlying darker sandy layer. The top layer had a larger hydraulic conductivity (K_S_) (sample size n = 2, mean = 1.61*10^–4^ m/s, range = 1.12*10^–4^–3.71*10^–4^ m/s) than the underlying layer (n = 3, mean K_S_ = 4.5*10^–5^ m/s, 9.26*10^–6^–1.16*10^–4^ m/s) (Supplementary Table T 1). Chemical analysis revealed suboxic to anoxic conditions in porewater at 25 cm depth and oxic surface water throughout the sampling campaign. Nutrient availability was elevated in surface water and pore water as indicated by nitrate and SRP (soluble reactive phosphorus) concentrations (Supplementary Figure S 1).

### Fluxes in surface water and the hyporheic zone

**Figure 1:**
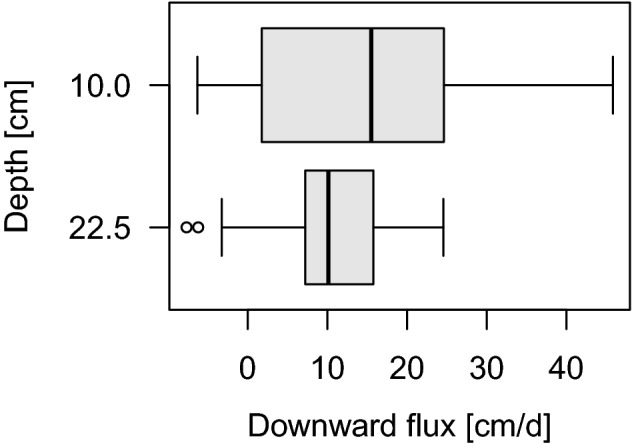
Seepage flux directions and velocities in 10.0 and 22.5 cm sediment depth calculated by the VFLUX 2.0 model using time series of temperature depth profiles (method: McCallum).

Daily WWTP-induced fluctuations of discharge and electrical conductivity (EC) in surface water (SW) were observed at our two sampling sites (Supplementary Figure S 2) which were both downstream of the WWTP. These fluctuations were delayed at the downstream site (SW_down_) compared to the site located 850 m upstream (SW_up_) of SW_down_ (see Methods and Fig. 5). Flow time between SW_up_ and SW_down_ obtained by cross-correlation was 5.9 h (CCF value = 0.907). Surface water flow velocities at SW_down_ ranged between 0.12–0.17 m/s (n = 10, mean = 0.15). Flow time calculated with that range of velocities and the length of the surface water stretch resulted in 1.4–1.9 h. Daily temperature fluctuations of pore water at the downstream site (PW_down_) (Supplementary Figure S 2) were used as input data for the model VFLUX to calculate seepage fluxes. VFLUX results indicated downward fluxes in all sediment depths with higher fluxes and variation at 10 cm sediment depth and lower fluxes and variation in 22.5 cm depth (Fig. 1). Mean velocities (n = 39, in 10 cm: 15.22 cm/d, in 22.5 cm: 10.53 cm/d) lead to a mean infiltration time of 1.93 days from surface water to 25 cm depth in the hyporheic zone.

### Trace organic compounds

**Figure 2:**
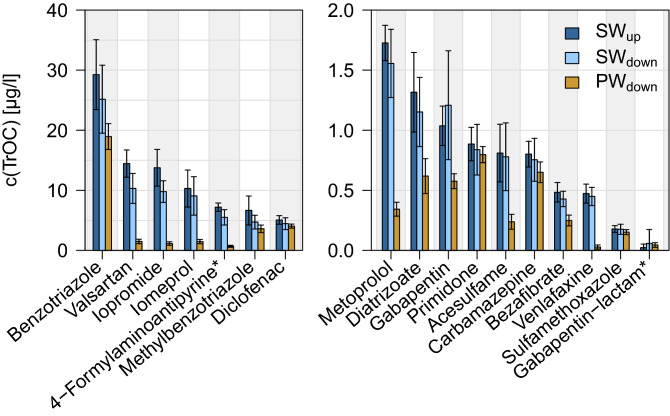
Mean TrOC concentrations at the upstream surface water sampling site (SW_up_), the downstream surface water sampling site (SW_down_) and the downstream pore water sampling site (PW_down_, in 25 sediment depth) (n = 17 (15 for SW_up)_, every 3 h over 48 h) . Graph split in two concentration ranges (right and left panel). Error bars indicate the standard deviation, * indicates transformation products.

Concentrations of TrOCs in individual surface water and pore water samples taken every three hours for 48 h ranged between 0.0 µg/l for gabapentin-lactam and 40.6 µg/l for benzotriazole. Mean concentrations (Fig. 2) of 13 out of 17 TrOCs differed stronger between SW_down_ and PW_down_ than between SW_up_ and SW_down_. Mean TrOC concentrations were decreased between the two respective sampling points on average by 11.7% in surface water (n = 16 (without gabapentin-lactam), range = − 16.5%–29.2%) and by 49.4% in the hyporheic zone (n = 17, 4.9–93.8%). If the transformation product gabapentin-lactam, which was not attenuated but formed, was also taken into account for surface water, the mean decrease of TrOC concentrations in surface water was 0.1% instead of 11.7%. Standard deviations of time series were smaller in pore water than at both surface water sites.

### Attenuation rates of TrOCs

Attenuation per meter flow distance was higher in the hyporheic zone with a mean of 9.723 µg/l/m (n = 17, range = 0.096–35.308 µg/l/m) than in surface water (mean = 0.001 µg/l/m, n = 17, range = 0.000–0.005 µg/l/h). In contrast, attenuation rates per hour residence time were on average slightly higher in surface water (mean = 0.180 µg/l/h, n = 17, range = − 0.029–0.702 µg/l/m) than in the hyporheic zone (mean = 0.053 µg/l/h, n = 17, range = 0.000–0.196 µg/l/m) (Supplementary Table T 2 and T 3).

### Dissolved organic matter

**Figure 3:**
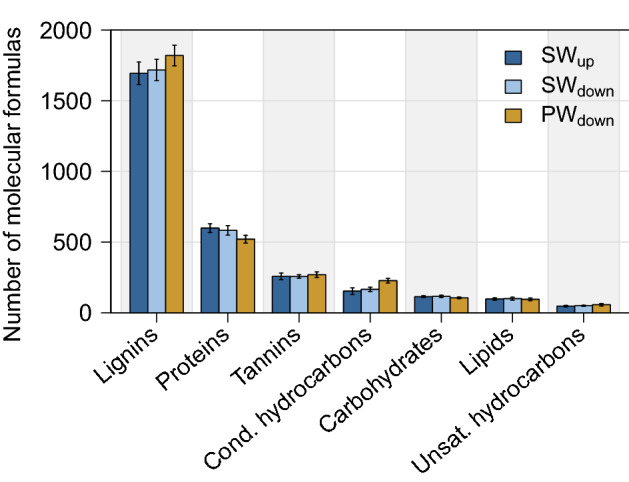
Number of molecular formulas grouped by DOM compound classes in surface water (SW) and pore water (PW) at upstream (_up_) and downstream (_down_) sites (n = 17, every 3 h over 48 h)Error bars indicate the standard deviation.

DOC concentrations in SW_up_ and SW_down_ averaged at 13.0 mg/l (n = 15, every 3 h over 48 h, range = 11.2–15.3 mg/l) and 12.2 mg/l (n = 17, range = 10.7–16.5 mg/l), respectively. DOC in pore water had an average concentration of 9.4 mg/l (n = 17, range = 8.3–11.6 mg/l). DOC concentrations were used as a minimum indicator for DOM concentrations as all DOC is included in DOM. The number of molecular formulas matching to DOM compound classes ranged between 39 for unsaturated hydrocarbon-like and 1833 for lignin-like formula in individual samples. H:C ratios decreased from SW_up_ to SW_down_ to PW_down_. Van Krevelen diagrams showing O:C versus H:C ratios of all detected molecules grouped by DOM classes, as means per sample, and for molecules which appeared or disappeared between sites are depicted in Supplementary Figures S 3–5. Changes in the mean number of molecular formulas of each DOM compound class (Fig. 3) were larger in the hyporheic zone (mean = 12.4%, n = 7, range: 5.0–36.8%) than in surface water (mean = 3.5%, n = 7, 0.3–8.4%). Numbers of molecular formulas of rather bioavailable compound classes (carbohydrate-, lipid-, protein-likes) decreased from SW_up_ to SW_down_ to PW_down_ while numbers of molecular formulas of more stable compounds (condensed hydrocarbon-, lignin-, tannin-, unsaturated hydrocarbon-likes) increased.

### Regression models for the relationship between chemistry and discharge

Quantile–Quantile plots and Shapiro–Wilk tests indicated normal distribution of residuals for all linear regression models except for the following substances at the upstream site: gabapentin-lactam (using discharge and EC as the independent variable); and at the downstream site: iomeprol (discharge, EC), gabapentin (discharge, EC), gabapentin-lactam (discharge, EC), primidone (discharge) and carbohydrate-like substances (EC). In these cases of non-normally distributed residuals, no correlation of the substance with discharge or EC was significant. 34 of the 38 linear regression models with normally distributed residuals did not have an R^2^ > 0.33 with p < 0.01 while four models had (Table 1).

**Table 1:** TrOCs and DOM compound classes for which linear regression with discharge or EC had an R^2^ > 0.33 and p-value < 0.01, classified by surface water sampling site.

Independent variable	Dependent variable in SW_up_ (R^2^, p-value)	Dependent variable in SW_down_ (R^2^, p-value)
Discharge	–	–
EC	Diatrizoate (0.57, 0.001) Iopromide (0.50, 0.003) Benzotriazole (0.49, 0.004)	Condensed hydrocarbons (0.47, 0.002)

Fluctuations in EC at the upstream site could explain between 49–57% of the fluctuations of diatrizoate, iopromide and benzotriazole (Table 1). EC represented substance transport which is dependent on discharge but is delayed compared to it. Therefore, significant correlations between concentrations of the TrOCs mentioned in SW_up_ could result from collinearity of the substances with delayed substance transport. This is of no concern for this study as we are only looking at relationships between TrOCs and DOM compound classes, but not between TrOCs themselves. In SW_down_, no potential collinearity with discharge or EC for correlating the substances with each other was found as no or only one substance was influenced by discharge or EC fluctuations.

### Correlation between TrOCs and DOM

**Figure 4:**
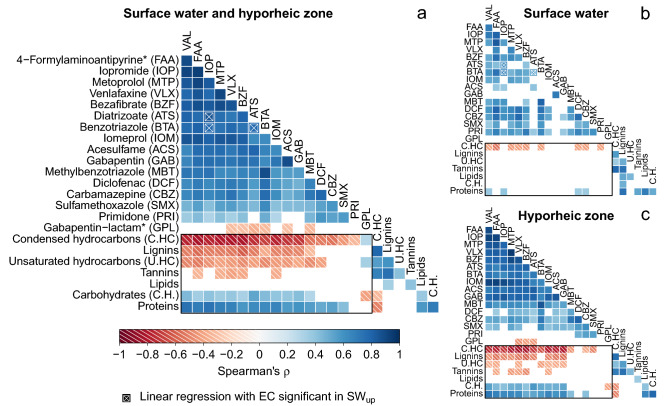
Correlation between TrOC concentrations and number of molecular formulas in DOM compound classes (black rectangle). Including (**a**) all data (SW_up_, SW_down_ and PW_down_), (**b**) only surface water data (SW_up_ and SW_down_) and (**c**) only data for the hyporheic zone at the downstream site (SW_down_ and PW_down_). Only significant correlations with p > 0.05 are shown. Correlations in SW_up_ potentially due to collinearity with EC, which serves as a proxy for delayed substance transport, are marked with an X on top of the rectangle. * indicates transformation products , VAL = valsartan.

Most and strongest correlations between TrOCs concentrations and numbers of molecular formulas in DOM compound classes were found when including all sampling sites (Fig. 4a). Molecular composition of three out of four DOM compound classes which are considered to be poorly biodegradable (condensed hydrocarbon-, lignin-, unsaturated hydrocarbon-likes) showed significant negative correlations (ρ = −0.31–0.82, p = 1.18*10^–10^–2.75*10^–2^) with at least 12 out of 17 TrOCs. In contrast, the molecular composition of the more labile compound classes carbohydrate-likes and protein-likes correlated positively (ρ = 0.29–0.75, p = 6.89*10^–10^–4.26*10^–2^) with 12 out of 17 TrOCs.

In surface water (Fig. 4b), significant correlations with 9 of the 17 TrOCs were found for condensed hydrocarbon-likes (ρ = −0.39–0.52, p = 2.07*10^–3^–2.80*10^–2^) and 4 out of 17 TrOCs for protein-likes (ρ = 0.35–0.39, p = 3.21*10^–2^–4.86*10^–2^). These correlations were weaker than the corresponding ones including all sampling sites. In contrast, correlations using only hyporheic zone samples, i.e. SW_down_ and PW_down_ samples (Fig. 4c), differed only slightly in strength and number of significant correlations from those including all data. Therefore, strong correlations including all data were mainly induced by the strong relationships in the hyporheic zone. Generally, correlations between rather persistent TrOCs (primidone, sulfamethoxazole, diclofenac, and carbamazepine) and DOM compound classes were weaker or not significant compared to TrOCs which underwent attenuation.

## Discussion

The relationship between changes in DOM composition and TrOC attenuation in a river and its hyporheic zone is still largely unknown. Previous laboratory studies^9–12,23,25^ observed a range of relationships with contradicting directions. With our sampling campaign, we can support or reject our two hypotheses:

Hypothesis (1): TrOC attenuation coincides with changes in DOM.

In the side channel “Rechter Randgraben” of the eutrophic^32^ River Erpe, we found positive or negative correlations between TrOC concentrations and the number of molecular formulas in DOM compound classes which are biodegradable or more recalcitrant, respectively (Fig. 4). Abundance and strength of correlations were larger in the hyporheic zone than in surface water. These correlations indicate that processes responsible for the turnover of bioavailable DOM either work very similar or are linked to processes of TrOC attenuation.

First, we tested for a relationship between discharge and chemical composition of surface water at our field sites to exclude the possibility of colinearity. In the main channel of the River Erpe, a relationship between most TrOC concentrations and discharge was observed previously^31^. In contrast, our findings do not indicate a relationship between TrOC concentration and discharge in the side channel, while daily EC fluctuations are not lost. Flow time between surface water sites derived from these EC fluctuations would lead to nearly stagnant surface water which is not the case at the study sites. In contrast, flow time calculated with distance and measured flow velocities at the downstream site was 3.5 times faster. The discrepancy between calculated flow times based on these two methods indicated that there must have been sections of slower flow velocities and transient storage in parts of the river stretch. After branching off from the main river, the side channel flows through a small, vegetated pond. Further downstream, water is dammed up in front of a pipe. These areas might have increased temporal retention of TrOCs and DOM. Dispersion, sorption and desorption could have further dissipated the initial relationship between substances and discharge. As a result of the dissipation of diurnal fluctuations of TrOCs, DOM and discharge, correlations between these compounds are unlikely to originate from collinearity with discharge.

FTICR-MS was used to analyse DOM. It is a high resolution method to examine DOM on a molecular scale. While this method does not result in DOM concentrations, it allowed us to observe qualitative changes in molecular composition of DOM. However, not all DOM compounds can be analysed with FTICR-MS. Non-ionizable molecules are excluded as they are not detectable using FTICR-MS^34^. Furthermore, only a certain proportion of DOM can be extracted with solid phase extraction (SPE) during sample preparation^35^. The priority pollutant (PPL) extraction cartridge we used allows for an efficient isolation of DOM using SPE^36^. In comparison to other extraction sorbents, PPL also leads to a molecular composition in the prepared sample which is closest to the one in the original sample^35^. Nevertheless, DOM compounds with higher molecular weight or low O:C ratio with limited polarity^35^ tend to be lost during the analytical process. This leads to an underestimation of molecular diversity. As all samples were treated equally, similar losses of molecular formulas were likely and comparability between samples should not have been affected.

Bioavailability of DOM was likely not limited by nutrient shortage^28^ at the study site due to elevated nutrient loads. The generally rather stable DOM composition (Fig. 3) could have resulted from an already high removal of biodegradable DOM during wastewater treatment^37^. As surface water was oxic, DOM availability and TrOC attenuation in surface water could not have been oxygen limited^21,29^, either. In contrast, this might have been the case for suboxic to anoxic pore water at sampling depth. However, changes in DOM and TrOCs were higher in the hyporheic zone than in surface water. As attenuation of most TrOCs is redox sensitive^21^, the major proportion of TrOC attenuation might have already occurred in the upper, supposedly oxic part of the sediment instead of along the whole hyporheic flow path. We conclude that bioavailability of DOM was probably not limited by external factors but was presumably rather dominated by its already high proportion of recalcitrant compounds. TrOC attenuation might have been oxygen-limited at pore water sampling depth.

Changes in DOM composition between sites can result from biodegradation, sorption, photosynthesis or addition of compounds. DOM compounds might have entered the stream along the flow path, e.g. with leaf litter and wood debris. This might explain increasing numbers of lignin-like molecules along flow paths. In surface water, photodegradation possibly attenuates photosensitive TrOCs like gabapentin^17^ or diclofenac^16^ and DOM compounds^38^. During our sampling campaign, gabapentin and diclofenac did not decrease in surface water. For DOM, sunlight can result in degradation of recalcitrant compounds to more labile molecules^39,40^ or in alteration of labile compounds to more stable substances^41^. In a previous study, semi labile DOM was more prone to photodegradation than labile DOM^42^. We observed a decrease in molecular diversity of bioavailable DOM compound classes while less bioavailable DOM molecular diversity increased in surface water. A high proportion of shaded surface area of the stream might have reduced the photodegradation potential. It was also dark half of the 48 h even though weather was sunny throughout daytime. Overall, it is reasonable to assume that photodegradation did not play a major role during our sampling campaign while natural material entering the stream might have.

In the present study, possibilities to analyse the effect of sorption are limited. While sorption of TrOCs to hyporheic sediment is either negligible^43^ or only influences some of the examined TrOCs (venlafaxine^44^, metoprolol and bezafibrate^18^), it can considerably affect DOM availability^28^. TrOCs for which substantial sorption is possible (venlafaxine, metoprolol, bezafibrate) are amongst the TrOCs which correlate with most DOM compound classes in the hyporheic zone. As some other TrOCs which are not prone to sorption also show similar correlations, sorption was not clearly distinguishable from biotransformation but must have been either small or parallel to biodegradation. For a profound discussion on potential attenuation pathways for TrOCs: See Supplementary Discussion.

Biodegradation of DOM leads to a more stable DOM composition^45^. In our study, we observed an increase in rather stable DOM molecular formula in surface water and the hyporheic zone. Furthermore, the decrease of H:C ratios from SW_up_ to SW_down_ to PW_down_ indicated an increase in double bonds and ring structures and thus a decrease in bioavailability^46^.

Composition and concentration of DOM in turn can impact attenuation of TrOCs. In previous soil column experiments, higher attenuation of diclofenac occurred under the presence of refractory DOC compared to no DOC^47^ or under lower than higher BDOC concentrations^13^. Likewise, we observed strong negative correlations between TrOCs and molecular numbers of rather stable DOM compound classes in the hyporheic zone. On the contrary, Hellauer et al.^47^ observed DOC-independent attenuation of benzotriazole, venlafaxine, metoprolol, sulfamethoxazole, iopromide and gabapentin. In contrast, we found positive correlations for at least two bioavailable DOM compound classes and negative correlations for at least two more stable DOM compound classes with those TrOCs except sulfamethoxazole. Another soil column study observed no effect of DOC with different bioavailability on the attenuation of TrOCs (carbamazepine, diclofenac, metoprolol and sulfamethoxazole, amongst others)^48^. However, in the present study, the direction of correlations between DOM compound classes and TrOCs differed with bioavailability of the DOM compound class. Furthermore, positive correlations between TrOCs and molecular diversity of bioavailable DOM contradict a general prevention of TrOC attenuation by the presence of bioavailable DOM, as observed in some previous studies^11,12^. However, concentrations of bioavailable and recalcitrant DOM would be needed to verify that assumption.

Previous studies suggest microbial co-metabolism as the link between TrOCs attenuation and DOM turnover^49^. Tang et al.^50^ observed higher TrOC attenuation under higher concentrations of complex DOM and associated that with microbial co-metabolism. While heterotrophic microbes can attenuate TrOCs either metabolically or co-metabolically, only a metabolic mechanism was observed for autotrophic microbes^49^. A previous study at the main River Erpe^31^ assumed that an observed parallel decrease of TrOCs and DOC availability during infiltration was due to coupling of the processes via microbial co-metabolism. This is in accordance with positive correlations between TrOCs and molecular diversity of bioavailable DOM compound classes we found in the hyporheic zone but not in surface water. While we observed strong correlations between TrOCs and DOM compound classes, individual substances within a compound class might behave differently from its class. Therefore, our results from correlation analysis indicate similar or linked turnover processes of TrOCs and bioavailable DOM as DOM compound classes, not as individual formulas.Correlations between TrOC concentrations and molecular formulas in DOM compound classes were strong and abundant in the hyporheic zone of this urban stream. In contrast, correlations in surface water were only observed for some TrOCs with protein (positive) or condensed hydrocarbons (negative). For surface water, the hypothesis of simultaneous attenuation of TrOCs and DOM compositional change therefore has to be rejected for all DOM compound classes except proteins. In contrast, our results supported this hypothesis for the hyporheic zone.

Hypothesis (2) (Simultaneous) Turnover of TrOCs and DOM mainly in hyporheic zone.

We observed a higher decrease in TrOC concentrations and stronger changes in molecular diversity of DOM compound classes in the hyporheic zone than in surface water. Similarly, DOC concentrations, which served as a minimum proxy parameter for DOM concentrations, also decreased stronger in the hyporheic zone than in surface water. TrOC concentrations were in the same order of magnitude as previous studies at the main channel^21,31,33^ (see Supplementary Discussion).

To attribute changes in hyporheic water chemistry to processes along the infiltration pathway and not to dilution by upwelling groundwater, seepage flux directions and velocities were calculated. VFLUX results indicated losing conditions. Larger variation and intensity in fluxes in 10 cm compared to 22.5 cm depth could be explained by hyporheic exchange fluxes in the upper part of the streambed sediment. These hyporheic exchange fluxes signify flow paths through the river bed originating and ending in surface water. Calculating seepage velocities with VFLUX is restricted by environmental and method limitations. Irvine et al.^51^ suggests a minimum time series comprising seven days of 24 h diurnal temperature signals. Due to changes in weather conditions, vertical seepage fluxes could only be calculated for a period of three days prior to the sampling campaign. Hence, calculated fluxes only give an indication for general seepage flux direction and magnitude. Determined fluxes were in accordance with the assumption of downward flux for calculating attenuation of TrOCs and change in DOM compound classes from surface to pore water.

Biotransformation is usually seen as the main attenuation pathway for many TrOCs in previous studies^11,52,53^ and is also crucial for DOM turnover^54^. The hyporheic zone is associated with elevated microbial activity and biogeochemical cycling^14^. This is in accordance with stronger TrOC attenuation and changes in DOM molecular diversity we observed in the hyporheic zone compared to surface water. Overall, we can assume that there was a high potential for biotransformation of TrOCs and DOM in the hyporheic zone of the side channel.

However, total TrOC and DOM turnover might have been limited by little hyporheic exchange. TrOC concentrations were attenuated strongly (Fig. 2) along the hyporheic flow path of 25 cm, while TrOCs decreased only slightly in the studied surface water stretch of 850 m. If hyporheic exchange would have been more intense along the surface water stretch, a larger proportion of TrOCs would have already been attenuated in the water reaching the downstream site.

Spatial TrOC attenuation rates in the hyporheic zone were on average 4.5 orders of magnitude higher than in surface water indicating that the hyporheic zone is a hotspot for TrOC turnover. In contrast, temporal attenuation rates of TrOCs were on average 3.4 times faster in surface water than the hyporheic zone indicating that the hyporheic zone is no hotspot for TrOC turnover. However, attenuation rates for surface water include attenuation due to hyporheic exchange. It is not possible to distinguish between attenuation occurring in surface water or occurring through hyporheic exchange along the stretch. In addition, attenuation rates of TrOCs in the hyporheic zone were calculated for the flow path to 25 cm depth with suboxic-anoxic conditions. Although, it is likely that most attenuation already occurred in the oxic part of the sediment i.e. the upper few centimenters^21^. While the potential for TrOC and DOM turnover is high in the hyporheic zone of the side channel, limited hyporheic flux as indicated by slow seepage fluxes (Fig. 1) might restrict the absolute turnover potential in the hyporheic zone. Therefore, the potential for TrOC attenuation and DOM turnover is likely to be higher in streams with increased hyporheic exchange.

Both, the attenuation of TrOCs and changes in molecular composition of DOM compound classes as well as correlations between these substances were stronger along the 25 cm long hyporheic flow path than along the 850 m long surface water reach. Therefore, the hypothesis that changes in DOM and TrOCs would mainly occur in the hyporheic zone was supported. Furthermore, restricted turnover of DOM and TrOCs in surface water compared to increased attenuation in the hyporheic zone indicated limited hyporheic exchange along the studied section of the stream. This assumption was supported by modelled limited downward flux.

Although strong correlations between TrOCs and DOM compound classes were found, they do not allow for concluding a causal relationship. Instead, these findings potentially indicate a connection between TrOCs and DOM turnover, possibly through co-metabolism. For future studies, disentangling the functional relationship between TrOCs, DOM, microbial metabolism and sorption would be of high interest.

We conclude that the attenuation of TrOCs and change in molecular composition of easily biodegradable DOM in an urban stream can occur simultaneously. In contrast, poorly biodegradable DOM compounds are not degraded coinciding with TrOCs attenuation, presumably because of complex molecular structures or because the energy gained from their metabolization is insufficient to enable microbial growth and co-metabolism of TrOCs. Contrary to some previous studies on the one hand and in compliance with some other studies on the other hand, we did not find indicators for the prevention of TrOC attenuation by the presence of (B)DOM . The hyporheic zone of a stream has a high potential for attenuation processes but restricted hyporheic exchange limits the attenuation of TrOC concentrations and DOM turnover in a stream.

## Methods

### Site description and experimental setup

**Figure 5:**
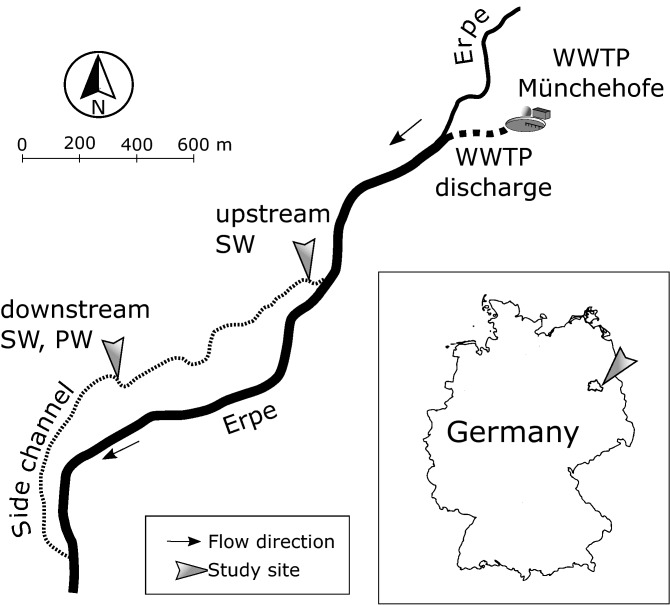
Map of the study site. WWTP: wastewater treatment plant. River compartments from which samples have been taken are indicated (SW: surface water, PW: pore water).

The River Erpe is an eutrophic^32^ urban lowland river in the East of Berlin. It is highly influenced by daily fluctuating effluents of the wastewater treatment plant Münchehofe and consists of up to 80% of treated wastewater^32^. As a result, discharge and electrical conductivity (EC) also fluctuate daily. A side channel (“Rechter Randgraben”) with a mean discharge of 25 l/s branches off the River Erpe 700 m after the treatment plant’s outlet (Fig. 5).

At this side channel, we have chosen an upstream site (52.479659 N, 13.635827 E) and a second site (52.476437 N, 13.625765 E) 850 m downstream of the upstream site (Fig. 5). An automatic sampler took 250 ml surface water samples at the upstream site (“SW_up_”) for every sampling time point. The experimental setup at the downstream site followed the sampling protocol of the Worldwide Hydrobiogeochemistry Observation Network for Dynamic River Systems (WHONDRS^57^). At every sampling time point, surface water samples (60 ml) were collected manually with a syringe and tubing fixed in the water column (“SW_down_”). In addition, we took pore water samples (60 ml) at the downstream site (“PW_down_”) in 25 cm sediment depth with three stainless steel rods used as replicates (see Supplementary Methods). This depth was assumed to comprise oxic and anoxic sediment zones and was chosen to account for redox sensitivity of TrOC attenuation^21,58^. Samples were taken every three hours over a time period of 48 h in September 2018. Due to a device failure, SW_up_ samples were not taken at hours 15 and 18. For details on sediment analysis: See Supplementary Methods.

### Fluxes in surface water and the hyporheic zone

Water pressure and EC were measured with CTD Divers (Van Essen Instruments B.V., Netherlands) in all compartments from one week before to one week after the sampling campaign in one-minute intervals (more details see Supplementary Methods).We measured flow velocity and discharge at the downstream site with a handheld flow meter (MF pro, OTT Hydromet, Loveland, USA) ten times covering the whole range from minimum to maximum water level. A rating curve was fitted to the point measurements by combining discharge and water pressure data. Time series of discharge were calculated from the time series of pressure data using this rating curve.

Flow time between SW_up_ and SW_down_ was calculated by cross correlating EC time series (21.09.2018 20:00 h–02.10.2018 20:00 h) of these two sites. A cross correlation function (CCF) value of 1 (or -1) shows maximum positive (negative) strength and a CCF value of 0 indicates no correlation at all. Due to a malfunction of our setup resulting in sediment in the measuring chamber, EC could not be continuously measured in pore water and therefore not be used to calculate hyporheic infiltration time.

Additionally, temperature was measured in surface water and 5, 15, and 30 cm sediment depth at the downstream site with a multilevel temperature lance (Umwelt- und Ingenieurtechnik GmbH Dresden, Germany) every minute. The MATLAB based program VFLUX 2.0^59^ with a heat transport model introduced by McCallum et al.^60^ was applied to calculate vertical seepage velocities within the hyporheic zone based on temperature time series in the streambed (further information in Supplementary Methods). Sensor combinations of 5 and 15 cm (sensor pair 1), as well as 15 and 30 cm depth (sensor pair 2) were used to calculate fluxes in 10 and 22.5 cm sediment depth at an interval of two hours.

Due to a severe change in weather conditions disturbing the use of temperature as a tracer, only the time period from 23.09.2018 16:00 h to 26.09.2018 21:00 h could be used to run the model. Fluxes in both sediment depths were averaged to a general flow direction and velocity. To obtain mean seepage velocity, Darcy velocity calculated by VFLUX 2.0 was divided by the mean porosity of the sediment (0.39, n = 6, range = 0.32–0.45).

Flow time T_Flow_ (d) from surface water to porewater in 25 cm depth was calculated (Eq. (1)) with $\Delta x$ (cm) being the vertical distance between given points (streambed surface = 0 cm, sensor 1 = 5 cm, sensor 2 = 15 cm, sampling depth = 25 cm), and $\overline{v}$ (cm d^-1^) being the calculated mean seepage velocity between temperature sensor pairs.

1 $${\text{T}}_{{{\text{Flow}}}} = \frac{{\Delta {\text{x}}_{{{\text{Sensor}} 1 - {\text{Streambed}} }} }}{{\overline{v}_{{{\text{Sensor}}\,{\text{pair}} 1}} }} + \frac{{\Delta {\text{x}}_{{{\text{Sensor}} 2 - {\text{Sensor}} 1}} }}{{\overline{v}_{{{\text{Sensor}}\;{\text{pair}} 1}} }} + \frac{{\Delta {\text{x}}_{{{\text{Depth}}_{{25\;{\text{cm}}}} - {\text{Sensor}}2}} }}{{\overline{v}_{{{\text{Sensor}}\;{\text{pair}} 2}} }}$$

### Chemical analysis

Water samples were filtered with 0.2 µm (polyethersulfone Sterivex for FTICR-MS analysis or regenerated cellulose for all other analytes), acidified to a pH of 2 with 2 M HCl (where indicated with*), cooled in field and during transportation, and stored at − 18 °C until analysis. Samples were analysed at the Leibniz Institute of Freshwater Ecology and Inland Fisheries for nitrate and sulfate (ion chromatography, Metrohm 930 Compact IC Flex), ammonium and SRP (*segmented flow analyser Skalar SAN, Skalar Analytical B.V., Netherlands) as well as manganese (Mn^2+^) and iron (Fe^2+^) (*inductively coupled plasma optical emission spectrometry (ICP-OES) ICP iCAP 6000series, Thermo Fisher Scientific Inc.). DOC concentrations were analysed via NDIR after combustion (*TOC/TN Analysator, Shimadzu). 17 selected TrOCs were quantified using high-performance liquid chromatography coupled with tandem mass spectrometry (HPLC–MS/MS, TSQ Vantage, Thermo Fisher Scientific, USA) at the Chair of Water Quality Engineering at the Technische Universität Berlin (TUB). DOM data is part of the WHONDRS dataset^61^ and was analysed using a 12 T (12 T) Bruker SolariX Fourier transform ion cyclotron resonance mass spectrometer (FTICR-MS; Bruker, SolariX, Billerica, MA, USA) at the Environmental Molecular Sciences Laboratory in Richland, WA. Once peaks were picked using the Bruker DataAnalysis software and formulas were assigned using Formularity^62^, DOM was classified into seven compound classes based upon H:C, and O:C ratios^63^. As the FTICR-MS analysis does not allow for a quantitative approach, compound class data was analysed qualitatively: DOM composition was evaluated using the number of molecular formulas in every compound class. Further details on TrOCs and DOM analysis are available in the Supplementary Methods.

### Data analysis

To ensure that correlations between TrOCs concentrations and the number of molecular formulas in each DOM compound class did not result from mutual dependence on discharge fluctuations, their relationship to discharge was analysed. EC fluctuations were induced by discharge fluctuations but are delayed to them as pressure travels faster than advective transport. This makes EC fluctuations a proxy for transported substances. Therefore, linear regression between discharge or EC and each TrOC or DOM compound class was modelled. Residuals of each linear regression model were tested for normal distribution by visually comparing sample quantiles with theoretical quantiles and by conducting a Shapiro–Wilk test (significance level: p-value > 0.01). If residuals were not normally distributed, correlation was calculated using Spearman’s ρ (significance level: p-value < 0.01) instead. A substance was considered as independent from discharge if R^2^ of the model was < 0.33 (significance level: p-value < 0.01) or correlation was not significant.

The mean change $\overline{a}_{xDOM}$ of a DOM compound class $x$ on the whole DOM composition was calculated as the difference between the mean number of molecular formulas $n$ matched to a given compound class $x$ at the first site $\overline{n}_{x, in}$ and second site in flow direction $\overline{n}_{x, out}$, divided by $\overline{n}_{x,in}$ (Eq. (2)). Total mean change of all compounds was calculated averaging absolute values of all ${\overline{\text{a}}}_{{{\text{xDOM}}}}$ as DOM molecules can be transformed into other compound classes during their degradation process.

2 $${\overline{\text{a}}}_{{\text{xDOM }}} { = }\frac{{\overline{n}_{x, in} - \overline{n}_{x, out} }}{{\overline{n}_{x,in} }}$$

Mean attenuation $\overline{a}_{TrOC}$ of a TrOC (µg l^-1^) was calculated similar to $\overline{a}_{xDOM}$ using the mean concentrations $\overline{c}_{x}$ (µg l^-1^) of the TrOC at two sites and normalizing the difference to $\overline{c}_{x, in}$ (Eq. (3)).

3 $${\overline{\text{a}}}_{{{\text{xTrOC}}}} = \frac{{{\overline{\text{c}}}_{{{\text{x}}, {\text{in}}}} - {\overline{\text{c}}}_{{{\text{x}},{\text{out}}}} }}{{{\overline{\text{c}}}_{{{\text{x}},{\text{in}}}} }}$$

Temporal (${\text{r}}_{{\text{y}}}\left({\text{t}} \right)$, (µg l^-1^ h^-1^)) and spatial attenuation rates (${\text{r}}_{{\text{y}}} \left( {\text{s}} \right)$, (µg l^-1^ m^-1^)) per hour residence time t (h) (Eq. 4) and per meter flow distance s (m) (Eq. 5) were calculated for each TrOC and river compartment $y$ (SW_up_-SW_down_ and SW_down_-PW_down_). To identify which compartment is a hotspot and exhibits a higher potential for TrOC attenuation, these attenuation rates were compared between surface water and the hyporheic zone.

4 $${\text{r}}_{{\text{y}}} \left( {\text{t}} \right) = { }\frac{{{\overline{\text{a}}}_{{{\text{xTrOC}}}} }}{{\text{t}}}$$

5 $${\text{r}}_{{\text{y}}} \left( {\text{s}} \right) = { }\frac{{{\overline{\text{a}}}_{{{\text{xTrOC}}}} }}{{\text{s}}}$$

Simultaneous attenuation of DOM and TrOCs was analysed by correlating numbers of molecular formulas in each DOM compound class to TrOC concentrations (Spearman’s ρ, significance level = 0.05). Correlations were calculated including a) all samples, b) only surface water samples (SW_up_ and SW_down_) or c) only hyporheic zone samples (SW_down_ and PW_down_). For all data analysis, R version 3.6.2^64^ was used where not indicated differently.

## Supplementary Information

Supplementary Information

## Data Availability

All data on which this study is based except FTICR data are available via the Freshwater Research and Environmental Database (FRED) of the Leibniz Institute of Freshwater Ecology and Inland Fisheries, https://doi.org/10.18728/556.0. Peak-picked FTICR-MS data and various meta-data are accessible on the U.S. Department of Energy’s Environmental System Science Data Infrastructure for a Virtual Ecosystem (ESS-DIVE) via https://data.ess-dive.lbl.gov/view/doi:10.15485/1577260.

## References

[1] Walsh CJ (2005). The Urban stream syndrome: current knowledge and the search for A cure. Am. Benthol. Soc.

[2] Putschew A, Wischnack S, Jekel M (2000). Occurrence of triiodinated X-ray contrast agents in the aquatic environment. Sci. Total Environ..

[3] Troger R, Kohler SJ, Franke V, Bergstedt O, Wiberg K (2020). A case study of organic micropollutants in a major Swedish water source - Removal efficiency in seven drinking water treatment plants and influence of operational age of granulated active carbon filters. Sci. Total Environ..

[4] Ruff M, Mueller MS, Loos M, Singer HP (2015). Quantitative target and systematic non-target analysis of polar organic micro-pollutants along the river Rhine using high-resolution mass-spectrometry–Identification of unknown sources and compounds. Water Res..

[5] Peralta-Maraver I, Posselt M, Perkins D, Robertson A (2019). Mapping micro-pollutants and their impacts on the size structure of streambed communities. Water.

[6] Malaj E (2014). Organic chemicals jeopardize the health of freshwater ecosystems on the continental scale. Proc. Natl. Acad. Sci. U.S.A..

[7] Petrie B, Barden R, Kasprzyk-Hordern B (2015). A review on emerging contaminants in wastewaters and the environment: Current knowledge, understudied areas and recommendations for future monitoring. Water Res..

[8] Gücker B, Brauns M, Pusch MT (2006). Effects of wastewater treatment plant discharge on ecosystem structure and function of lowland streams. J. North Am. Benthol. Soc..

[9] Lim M-H, Snyder SA, Sedlak DL (2008). Use of biodegradable dissolved organic carbon (BDOC) to assess the potential for transformation of wastewater-derived contaminants in surface waters. Water Res..

[10] Rauch-Williams T, Hoppe-Jones C, Drewes JE (2010). The role of organic matter in the removal of emerging trace organic chemicals during managed aquifer recharge. Water Res..

[11] Drewes JE (2014). Tuning the performance of a natural treatment process using metagenomics for improved trace organic chemical attenuation. Water Sci. Technol..

[12] Li D, Alidina M, Drewes JE (2014). Role of primary substrate composition on microbial community structure and function and trace organic chemical attenuation in managed aquifer recharge systems. Appl. Microbiol. Biotechnol..

[13] Alidina M, Li D, Ouf M, Drewes JE (2014). Role of primary substrate composition and concentration on attenuation of trace organic chemicals in managed aquifer recharge systems. J. Environ. Manag..

[14] Battin TJ, Besemer K, Bengtsson MM, Romani AM, Packmann AI (2016). The ecology and biogeochemistry of stream biofilms. Nat. Rev. Microbiol..

[15] Peralta-Maraver I, Reiss J, Robertson AL (2018). Interplay of hydrology, community ecology and pollutant attenuation in the hyporheic zone. Sci. Total Environ..

[16] Kunkel U, Radke M (2012). Fate of pharmaceuticals in rivers: deriving a benchmark dataset at favorable attenuation conditions. Water Res..

[17] Herrmann M, Menz J, Olsson O, Kummerer K (2015). Identification of phototransformation products of the antiepileptic drug gabapentin: Biodegradability and initial assessment of toxicity. Water Res..

[18] Riml J, Wörman A, Kunkel U, Radke M (2013). Evaluating the fate of six common pharmaceuticals using a reactive transport model: Insights from a stream tracer test. Sci Total Environ.

[19] Zhang X (2018). Removal of acidic pharmaceuticals by small-scale constructed wetlands using different design configurations. Sci Total Environ.

[20] Meierjohann A, Brozinski JM, Kronberg L (2016). Seasonal variation of pharmaceutical concentrations in a river/lake system in Eastern Finland. Environ. Sci. Process. Impacts.

[21] Schaper JL (2018). The fate of polar trace organic compounds in the hyporheic zone. Water Res..

[22] Asif MB, Hou J, Price WE, Chen V, Hai FI (2020). Removal of trace organic contaminants by enzymatic membrane bioreactors: Role of membrane retention and biodegradation. J. Membr. Sci..

[23] Maeng SK, Sharma SK, Abel CDT, Magic-Knezev A, Amy GL (2011). Role of biodegradation in the removal of pharmaceutically active compounds with different bulk organic matter characteristics through managed aquifer recharge: Batch and column studies. Water Res..

[24] Foulquier A, Mermillod-Blondin F, Malard F, Gibert J (2011). Response of sediment biofilm to increased dissolved organic carbon supply in groundwater artificially recharged with stormwater. J Soil Sediment.

[25] Onesios KM, Bouwer EJ (2012). Biological removal of pharmaceuticals and personal care products during laboratory soil aquifer treatment simulation with different primary substrate concentrations. Water Res..

[26] Hoppe-Jones C, Dickenson ER, Drewes JE (2012). The role of microbial adaptation and biodegradable dissolved organic carbon on the attenuation of trace organic chemicals during groundwater recharge. Sci Total Environ.

[27] Ide JI (2017). Spatial variations in the molecular diversity of dissolved organic matter in water moving through a boreal forest in eastern Finland. Sci. Rep..

[28] Marschner B, Kalbitz K (2003). Controls of bioavailability and biodegradability of dissolved organic matter in soils. Geoderma.

[29] Sobczak WV, Findlay S (2002). Variation in bioavailability of dissolved organic carbon among stream hyporheic flowpaths. Ecology.

[30] Harjung A, Sabater F, Butturini A (2018). Hydrological connectivity drives dissolved organic matter processing in an intermittent stream. Limnologica.

[31] Schaper JL (2019). Fate of trace organic compounds in the hyporheic zone: Influence of retardation, the benthic biolayer, and organic carbon. Environ. Sci. Technol..

[32] Lewandowski J, Putschew A, Schwesig D, Neumann C, Radke M (2011). Fate of organic micropollutants in the hyporheic zone of a eutrophic lowland stream: Results of a preliminary field study. Sci Total Environ.

[33] Jaeger A (2019). Spatial and temporal variability in attenuation of polar organic micropollutants in an urban lowland stream. Environ. Sci. Technol..

[34] Nebbioso A, Piccolo A (2013). Molecular characterization of dissolved organic matter (DOM): a critical review. Anal. Bioanal. Chem..

[35] Raeke J, Lechtenfeld OJ, Wagner M, Herzsprung P, Reemtsma T (2016). Selectivity of solid phase extraction of freshwater dissolved organic matter and its effect on ultrahigh resolution mass spectra. Environ. Sci. Process Impacts.

[36] Dittmar T, Koch BP, Hertkorn N, Kattner G (2008). A simple and efficient method for the solid-phase extraction of dissolved organic matter (SPE-DOM) from seawater. Limnol. Oceanogr.-Meth..

[37] Wang M, Chen Y (2018). Generation and characterization of DOM in wastewater treatment processes. Chemosphere.

[38] Wilske C, Herzsprung P, Lechtenfeld O, Kamjunke N, Tümpling W (2020). Photochemically induced changes of dissolved organic matter in a humic-rich and forested stream. Water.

[39] Wetzel RG, Hatcher PG, Bianchi TS (1995). Natural photolysis by ultraviolet irradiance of recalcitrant dissolved organic matter to simple substrates for rapidbacterial metabolism. Limnol. Oceanogr..

[40] Bertilsson S, Stefan LJ (1998). Photochemically produced carboxylic acids as substrates for freshwater bacterioplankton. Limnol. Oceanogr..

[41] Ward CP, Nalven SG, Crump BC, Kling GW, Cory RM (2017). Photochemical alteration of organic carbon draining permafrost soils shifts microbial metabolic pathways and stimulates respiration. Nat. Commun..

[42] Bowen JC, Kaplan LA, Cory RM (2020). Photodegradation disproportionately impacts biodegradation of semi-labile DOM in streams. Limnol. Oceanogr..

[43] Alidina M, Li D, Drewes JE (2014). Investigating the role for adaptation of the microbial community to transform trace organic chemicals during managed aquifer recharge. Water Res..

[44] Writer JH, Antweiler RC, Ferrer I, Ryan JN, Thurman EM (2013). In-stream attenuation of neuro-active pharmaceuticals and their metabolites. Environ. Sci. Technol..

[45] Kalbitz K (2003). Changes in properties of soil-derived dissolved organic matter induced by biodegradation. Soil Biol. Biochem..

[46] Wu Z, Rodgers RP, Marshall AG (2004). Two- and three-dimensional van Krevelen diagrams: a graphical analysis complementary to the kendrick mass plot for sorting elemental compositions of complex organic mixtures based on ultrahigh-resolution broadband fourier transform ion cyclotron resonance mass measurements. Anal. Chem..

[47] Hellauer K, Martínez Mayerlen S, Drewes JE, Hübner U (2019). Biotransformation of trace organic chemicals in the presence of highly refractory dissolved organic carbon. Chemosphere.

[48] Bertelkamp C (2015). The effect of feed water dissolved organic carbon concentration and composition on organic micropollutant removal and microbial diversity in soil columns simulating river bank filtration. Chemosphere.

[49] Tran NH, Urase T, Ngo HH, Hu J, Ong SL (2013). Insight into metabolic and cometabolic activities of autotrophic and heterotrophic microorganisms in the biodegradation of emerging trace organic contaminants. Bioresour. Technol..

[50] Tang K (2017). Influence of humic acid addition on the degradation of pharmaceuticals by biofilms in effluent wastewater. Int. J. Hyg. Environ. Health.

[51] Irvine, D., Lautz, L. K., Briggs, M. A., Gordon, R. P. & McKenzie, J. M. *VFLUX 2: Vertical fluid heat transport solver. VFLUX 2 documentation version 2.0.0, Copyright 2011, 2012: Ryan P. Gordon*, < https://hydrology.syr.edu/wp-content/uploads/2016/03/vflux2.zip, Access: June 9, 2020> (2015).

[52] Trinh T (2016). Seasonal variations in fate and removal of trace organic chemical contaminants while operating a full-scale membrane bioreactor. Sci. Total Environ..

[53] Rutere C, Knoop K, Posselt M, Ho A, Horn M (2020). Ibuprofen degradation and associated bacterial communities in hyporheic zone sediments. Microorganisms.

[54] Anderson TR (2019). Unified concepts for understanding and modelling turnover of dissolved organic matter from freshwaters to the ocean: the UniDOM model. Biogeochemistry.

[55] QGIS.org: QGIS Geographic Information System 3.16.0. QGIS Association, http://www.qgis.org (2020).

[56] The Inkscape Team: Inkscape 0.92.4. https://inkscape.org/release/0.92.4/windows/ (2019).

[57] Stegen, J. C. & Goldman, A. E. WHONDRS: A community resource for studying dynamic river corridors. *mSystems* **3,** 4, doi:10.1128/mSystems.00151-18 (2018). 10.1128/mSystems.00151-18 PMC6178584 30320221

[58] Redeker M, Wick A, Meermann B, Ternes TA (2018). Anaerobic Transformation of the Iodinated X-ray Contrast Medium Iopromide, Its Aerobic Transformation Products, and Transfer to Further Iodinated X-ray Contrast Media. Environ. Sci. Technol..

[59] Gordon RP, Lautz LK, Briggs MA, McKenzie JM (2012). Automated calculation of vertical pore-water flux from field temperature time series using the VFLUX method and computer program. J. Hydrol..

[60] McCallum AM, Andersen MS, Rau GC, Acworth RIA (2012). 1-D analytical method for estimating surface water–groundwater interactions and effective thermal diffusivity using temperature time series. Water Resour. Res..

[61] Wells JR (2019). WHONDRS 48 hour diel cycling study at the Erpe River Germany.. DOE Data Explor..

[62] Tolić N (2017). Formularity: Software for automated formula assignment of natural and Other organic matter from ultrahigh-resolution mass spectra. Anal. Chem..

[63] Kim S, Kramer RW, Hatcher PG (2003). Graphical method for analysis of ultrahigh-resolution broadband mass spectra of natural organic matter, the Van Krevelen diagram. Anal. Chem..

[64] R Core Team. R: A Language and Environment for Statistical Computing. (2019).

