# Supplementary material for: Simultaneous attenuation of trace organics and change in organic matter composition in the hyporheic zone of urban streams
Source: Sci Rep. 2021 Feb 18;11:4179. doi: 10.1038/s41598-021-83750-8 (PMC7892836; doi:10.1038/s41598-021-83750-8)
Supplement: Supplementary file 1 — Supplementary Information [file 41598_2021_83750_MOESM1_ESM.pdf]

Supplementary information

on

Simultaneous attenuation of trace organics and change in organic matter composition in the hyporheic zone of urban streams

Birgit M. Mueller*[1,2], Hanna Schulz[1,3], Robert E. Danczak[4], Anke Putschew[2], Joerg Lewandowski[1,3]

[1] Department Ecohydrology, Leibniz Institute of Freshwater Ecology and Inland Fisheries, Mueggelseedamm 310, 12587 Berlin, Germany

[2] Department of Environmental Sciences and Technology, Chair of Water Quality Engineering, Technische Universität Berlin, Straße des 17. Juni 135, 10623 Berlin, Germany

[3] Geography Department, Humboldt University of Berlin, Rudower Chausee 16, 12489 Berlin, Germany

[4] Earth and Biological Sciences Directorate, Pacific Northwest National Laboratory, 902 Battelle Blvd., Richland, WA 99352, United States of America

Results

Sediment characteristics, redox conditions and nutrient availability

Supplementary Table T 1: Sediment characteristics, given as mean; range. TC = total carbon, K_S = hydraulic conductivity

Sediment depth [cm]	Gravel [%]	Sand [%]	Silt and clay [%]	TC [wt%]	K_S [m/s], normed to 10°C
0 to 4-9<br>(n = 3 (Ks: 2))	13.7;<br>12.9 – 14.4	85.8;<br>84.8 - 86.8	0.5;<br>0.4 – 0.8	0.5;<br>0.1 – 0.9	$1.61*10^{-4}$;<br>$1.12*10^{-4} – 3.71*10^{-4}$
> 4-9<br>(n = 3)	3.8;<br>2.4 – 5.1	94.7;<br>93.1 – 96.6	1.5;<br>1.1 – 1.8	1.4<br>1.1 – 2.0	$4.5*10^{-5}$;<br>$9.26*10^{-6}-1.16*10^{-4}$

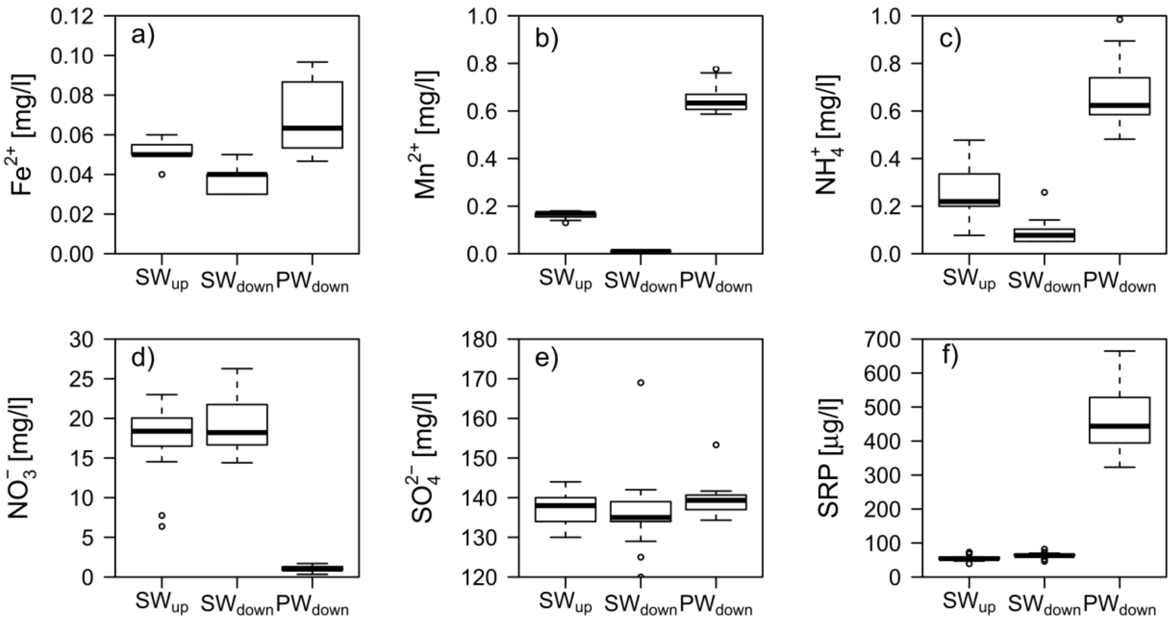

Supplementary Figure S 1: Redox indicators and nutrients a) dissolved iron, b) dissolved manganese, c) ammonia, d) nitrate, e) sulfate and f) soluble reactive phosphorus (SRP) in surface water (sampling sites SW_{up} and SW_{down}) and pore water (PW_{down}) of the hyporheic zone.

Lower concentrations of nitrate, higher concentrations of ammonia, dissolved manganese (Mn^{2+}) and dissolved iron (Fe^{2+}) but similar concentrations of sulfate in pore water compared to surface water (Supplementary Figure S 3) indicated suboxic to anoxic conditions in the sediment at 25 cm depth. Surface water samples showed oxic conditions throughout the sampling campaign. Elevated nitrate and soluble reactive phosphorus (SRP) concentrations indicate eutrophic conditions in surface and pore water.

Time series of temperature, discharge and electrical conductivity

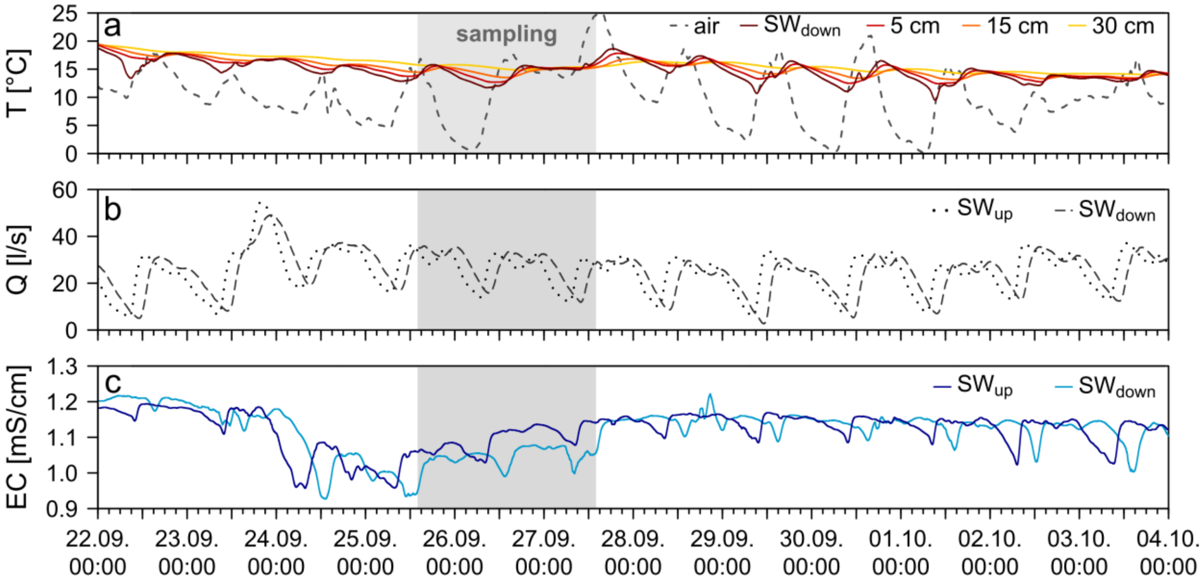

Supplementary Figure S 2: (a) Temperature (T), (b) discharge (Q) and (c) electrical conductivity (EC) in surface water at upstream and downstream site (SW_{up}, SW_{down}) and in pore water at downstream site (in the depth given in cm), displayed as the three hours rolling mean.

Attenuation rates of TrOCs per distance and residence time

Supplementary Table T 2: Change of TrOCs per meter flow distance between SW_{up} and SW_{down} and between SW_{down} and PW_{down}, given in µg $l^{-1}m^{-1}$.

	mean	ACS	ATS	IOM	IOP	GAB	FAA	BTA	SMX	MTP	PRI	MBT	VLX	GPL	CBZ	BZF	VAL	DCF
SW_{up} - SW_{down}	0.001	0	2.00E-04	0.001	0.005	-2.00E-04	0.002	0.005	0	2.00E-04	1.00E-04	0.002	0	0	1.00E-04	1.00E-04	0.005	7.00E-04
SW_{down} - PW_{down}	9.723	2.164	2.136	30.396	34.552	2.528	19.284	24.844	0.096	4.852	0.164	4.476	1.684	0.052	0.416	0.716	35.308	1.624

Supplementary Table T 3: Change of TrOCs per hour residence time between SW_{up} and SW_{down} and between SW_{down} and PW_{down}, given in µg $l^{-1}h^{-1}$.

	mean	ACS	ATS	IOM	IOP	GAB	FAA	BTA	SMX	MTP	PRI	MBT	VLX	GPL	CBZ	BZF	VAL	DCF
SW_{up} - SW_{down}	0.180	0.005	0.028	0.209	0.674	-0.029	0.287	0.694	3.00E-04	0.029	0.008	0.329	0.004	-0.006	0.008	0.010	0.702	0.105
SW_{down} - PW_{down}	0.053	0.012	0.012	0.164	0.187	0.014	0.104	0.134	5.00E-04	0.026	9.00E-04	0.024	0.009	3.00E-04	0.002	0.004	0.191	0.009

Change in DOM composition between sites

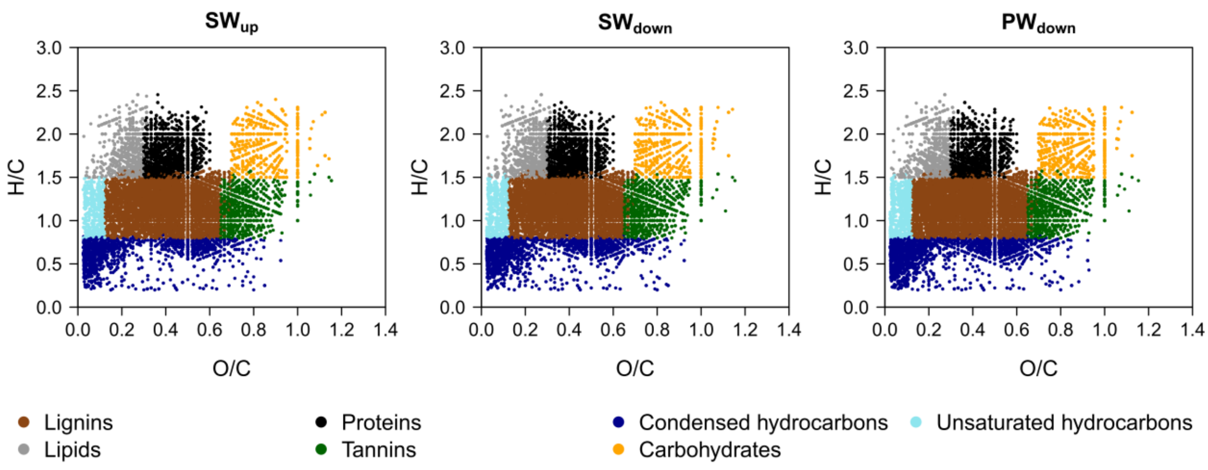

Supplementary Figure S 3: Van Krevelen diagrams of samples grouped per site. Displayed are only molecular formulas which have been matched with one of the seven DOM compound classes.

The composition of DOM at all three sampling sites looks similar when including all molecular formulas detected in every sample at the given site (Supplementary Figure S 3).

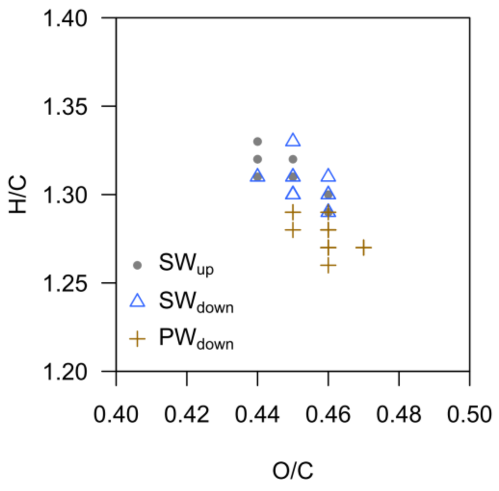

Supplementary Figure S 4: Van Krevelen diagram of mean O/C and H/C ratios per sample. Only ratios of molecules which matched one of the seven DOM compound classes displayed in Supplementary Figure S 3 were used to calculate averages.

When only comparing mean O/C ratios and H/C ratios in each sample (Supplementary Figure S 4), O/C ratios increase along the flow path from SW_{up} to SW_{down} to PW_{down}. In contrast, H/C ratios decrease along this flow path.

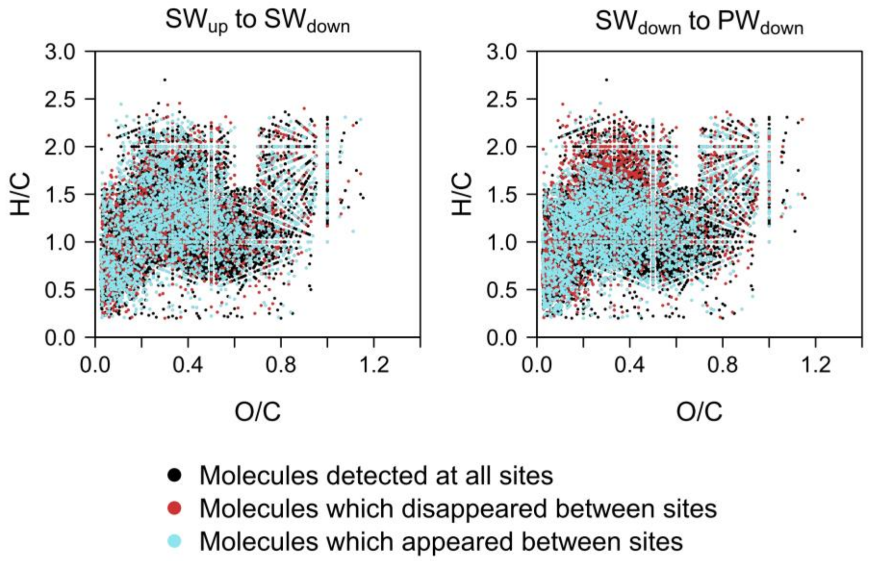

Supplementary Figure S 5: Comparison of molecular formulas unique to different sampling sites.

While DOM composition only changes slightly between the two surface water sites, especially the number of proteins and lipids decreases while the number of lignin-like molecular formulas increases between SW_{down} and PW_{down} (Supplementary Figure S 5).

Discussion

Attenuation of TrOCs

For many polar TrOCs, the main attenuation pathway is biotransformation[1-3]: e.g. for diclofenac[4], gabapentin[5], sulfamethoxazole[6], valsartan[7] or acesulfame[8]. While the first three compounds named were rather persistent in the River Erpe side channel, valsartan and acesulfame concentrations strongly decreased in the hyporheic zone. This indicates biotransformation as sorption has been reported to be negligible for both compounds[7,9]. In contrast, methylbenzotriazole is poorly biodegradable in the environment[10], which is in

accordance with its rather persistent behaviour in the present study. Writer, et al. [11] attributed the main attenuation of TrOCs in a river to interactions with the streambed sediments and biofilms. They identified sorption as the main attenuation process for venlafaxine despite being biotransformed under anaerobic conditions[12]. We observed a strong decrease of venlafaxine in the hyporheic zone in contrast to persistent behaviour in surface water. While Riml, et al. [13] found metoprolol and bezafibrate to be mainly affected by sorption, Kunkel and Radke [14] associated high attenuation of these TrOCs with biotransformation in the hyporheic zone. Both studies are in accordance with the decrease of bezafibrate and metoprolol found in the hyporheic zone of the River Erpe side channel.

Our ability to differentiate between sorption and biotransformation is limited. For TrOCs for which sorption has been identified as a relevant attenuation mechanism both sorption and biotransformation might have contributed to a decrease in their concentration. Retardation due to sorption was repeatedly found to be small or negligible either for all polar TrOCs[15,16] or for anionic or neutral compounds[4]. In contrast, sorption was often observed for cationic TrOCs[11]. In contrast, Schaper, et al. [17] observed substantial retardation attributed to reversible sorption for a variety of charges. Therefore, it is likely that sorption influenced at least metoprolol and venlafaxine, which occur as cations under the measured ambient pH value of 7.0-7.5 (anions: acesulfame, bezafibrate, diclofenac, sulfamethoxazole, valsartan; neutral: diatrizoate, benzotriazole, carbamazepine, 4-formylaminoantipyrine, gabapentin-lactam, iomperol, iopromide, methylbenzotriazole, primidone; zwitterion: gabapentin).

Sorption had to be taken into account for at least metoprolol and venlafaxine. In contrast, acesulfame had low sorption potential due to its negative charge and low retardation coefficient in the main River Erpe[17]. Similar to metoprolol and venlafaxine, acesulfame was only attenuated in the hyporheic zone. Correlations between DOM compound classes and metoprolol, venlafaxine or acesulfame in the hyporheic zone were similar for condensed

hydrocarbon-, lignin-, carbohydrate- and lipid-likes while metoprolol and venlafaxine also correlated with tannins, lipids and uncondensed hydrocarbons. Therefore, sorption of metoprolol and venlafaxine might have been either negligible or parallel to biodegradation of DOM compound classes but was not clearly distinguishable from biotransformation.

Gabapentin-lactam, a transformation product, increased in surface water and decreased in the hyporheic zone. In contrast, another transformation product, 4-formylaminoantipyrine, decreased in both compartments. While 4-formylaminoantipyrine is a human metabolite[18], degradation of gabapentin to its main transformation product gabapentin-lactam does not occur in the human body[19] but in freshwater-sediment systems via aerobic biotransformation[5]. Photolysis of gabapentin to gabapentin-lactam[20] might have also led to an increase of gabapentin-lactam in surface water. Nevertheless, there is no explanation for the highest mean in concentration of the parent compound gabapentin occurring at SW_{down} instead of SW_{up}. Possibly, this is an analytical error or the sampling interval was not frequent enough to cover the whole range of gabapentin concentrations with a relative standard deviation of 37.2 % at SW_{down}. Diclofenac was also reported to be photosensitive[14,21] but persistent to sorption and biotransformation[13]. We observed only small changes of concentration in surface water and pore water. A high percentage of shaded water surface area by riparian vegetation could have prevented photodegradation despite mostly sunny weather.

Comparison of TrOC concentrations to previous studies

For December 2015, Schaper, et al. [22] reported TrOC concentrations in a similar range in the surface water of the main channel of the River Erpe 150 m downstream of the branching of the side channel. Carbamazepine, gabapentin, metoprolol and acesulfame concentrations were twice as high while benzotriazole concentrations were half as high compared to concentrations found in the present study. Concentrations of bezafibrate, diclofenac, 4-formylaminoantipyrine, methylbenzotriazole, primidone, sulfamethoxazole and venlafaxine concentrations were comparable to the concentrations observed in the present study. Higher concentrations found by Schaper, et al. [22] might be due to the decreased

biodegradation capacity in the wastewater treatment plant under cold temperatures in winter[23]. In June 2016, another study at the River Erpe[17,24] found TrOC concentrations with greater deviations to the present study. Surface water concentrations had 1/3 lower diclofenac concentrations (30 cm PW: two times lower), 2/3 lower benzotriazole (four times lower), metoprolol (30 cm PW: 2/3 higher), sulfamethoxazole (20 times lower) and venlafaxine (six times higher) concentrations, ½ lower valsartan (twice as high) concentrations while acesulfame concentrations were three times higher (two times higher) and carbamazepine concentrations twice as high (twice as high). Other TrOCs, e.g. 4-formylaminoantipyrine, were found in similar concentration ranges in both studies.

Methods

Sampling method for pore water

Three stainless steel rods were placed in a line along flow direction in the middle of the channel with a distance of 2 m between two neighbouring rods. The rods (inner diameter 6.4 mm) were covered with a filter mesh sock over the screened area at the tip, pushed into the sediment and equipped with a Teflon suction line. Samples were taken by manually pulling 60 ml porewater with syringes attached to the suction line. Syringes and suction lines were flushed three times before taking a sample. Like surface water samples, pore water samples were taken every three hours over a 48 hour time period.

Sediment analysis

We took three sediment cores at the downstream site with 60 cm long PVC tubes in February 2019. From each core, the upper 5 cm of every visually distinguishable sediment layer were transferred to metal soil sample rings. Saturated hydraulic conductivity (K_S) was measured with a KSAT meter (KSAT, Meter Group, United States) and normalized to 10°C. Thermodynamic parameters needed for VFLUX 2.0 were determined with a thermal properties analyser (KD2 Pro, Decagon Devices Inc., Pullman, WA, USA).

In order to analyse porosity of the sediment, sediment samples were saturated with water when still in metal rings. Afterwards, saturated sediment samples were transferred to aluminium containers with

known mass and weighed. Sediment samples were then dried at 60 °C in a compartment drier for two days until mass remained constant. Dry samples were then weighed again after cooling to room temperature. Porosity n (%) was then calculated with Equation 1 where m_{sat} = mass of saturated sediment sample, m_{alu} = mass of aluminium container, m_{dry} = mass of dried sample, d_{H2O} = density of water and V_{ring} = volume of metal ring (= 250 cm³).

(1) $$n = \frac{(m_{sat} - m_{dry} - m_{alu}) / d_{H2O}}{V_{ring}}$$

For grain size distribution, dried sediment samples were sieved using a nested column of sieves on a mechanic sieve shaker. The sieves used had the following mesh opening sizes in mm from the top to the bottom of the nested sieves tower: 6.3, 2.0, 0.63, 0.18, 0.063, 0.02. After the sieving was completed, the mass of the sediment in each sieve was weighed and the proportion of each grain size was calculated by dividing the mass of sediment in each sieve by mass of the total sediment sample. Further sediment characterization included total carbon analysis (vario El III Element Analyzer, Elementar Analysensysteme, Germany) of dried, grinded and homogenized sediment. All parameters were averaged for each visually distinguishable layer.

Measurement of water pressure

In order to measure water pressure and EC, CTD Divers (Van Essen Instruments B.V., Netherlands) were used in all compartments. A diver for surface water was fixed to a metal pole which was installed next to the middle metal rod for sampling pore water with a distance of 10 cm. The metal pole was also used to fix the tubing for surface wate sampling in the water column.

Barometric pressure was measured using a TD logger (Van Essen Instruments B.V., Netherlands) installed in a shrub next to the stream at SW_{down}. Pressure data was corrected to atmospheric pressure by subtracting barometric pressure from water pressure data.

Trace organic compounds analysis

17 selected TrOCs were quantified in a total of 83 samples using high-performance liquid chromatography coupled with tandem mass spectrometry (HPLC-MS/MS, TSQ Vantage, Thermo Fisher Scientific, USA) at the Chair of Water Quality Engineering at the Technische Universität Berlin (TUB). For separation, the column XSelect HSS T3 (2.1 x 50 mm, particle size 2.5 µm, Waters, USA) was applied using a linear gradient from 98 % of ultrapure water to 100 % methanol (HPLC grade, J.T. Baker, USA), both acidified with formic acid, within 6 minutes. The compounds were ionized by pos/neg-electrospray ionization. After collision induced dissociation, two selected ion fragments showing the highest signal intensities were recorded for each compound. Internal calibration (0.01 – 55 µg/l) was applied for quantification using isotope-labelled standards (for acesulfame, benzotriazole, bezafibrate, carbamazepine, diclofenac, gabapentin, iomeprol, iopromide, methylbenzotriazole, primidone and sulfamethoxazole). Xcalibur 2.1 (Thermo Scientific, USA) was used for data processing and calibration curves were fitted to the measured calibration standard concentrations with a weighted (1/x) linear regression model. The limits of quantification were specified as the calibration concentration at which the less abundant iodized fragment had a signal to noise ratio smaller than 3.

FTICR-MS analysis

For the FTICR-MS analysis, surface water samples were split into three replicates. For hyporheic pore water, the three pore water samples taken in 25 cm sediment depth with three stainless steel rods at each time point were used as replicates. A total of 153 samples were analysed. Water samples (NPOC 0.33-0.99 mg C/L) were first acidified to pH 2 with 85% phosphoric acid and dissolved organic matter was extracted using PPL cartridges (Bond Elut, Agilent, CA, USA), following Dittmar, et al. [25]. Then, high-resolution mass spectra of the DOM were collected using a 12 Tesla (12T) Bruker SolariX Fourier transform ion cyclotron resonance mass spectrometer (FTICR-MS; Bruker, SolariX, Billerica, MA, USA) located at the Environmental Molecular Sciences Laboratory in Richland, WA. The resolution was 220K at 481.185 m/z. The FTICR-MS was equipped with a standard electrospray ionization (ESI) source, and data was acquired in negative mode with the needle voltage set to +4.4kV. Samples were directly injected into the instrument using a custom automated direct infusion cart that performed two offline blanks between each sample. Data were collected with an ion

accumulation time of 0.08 s from 100 m/z – 900 m/z at 4M. One hundred forty-four scans were co-added for each sample and internally calibrated using OM homologous series separated by 14 Da (–CH_2 groups). The mass measurement accuracy was typically within 1 ppm for singly charged ions across a broad m/z range (100 m/z - 900 m/z). Raw spectra were converted into lists of m/z values using Bruker Daltonik Data Analysis (version 4.2) and the FTMS peak picker module (signal-to-noise (S/N) ratio: 7; absolute intensity threshold: 100). Chemical formulae were assigned using an in-house software (Formularity; Tolić, et al. [26]) according to the Compound Identification Algorithm[27-29] based on the following criteria: S/N >7, and mass measurement error < 0.5 ppm, taking into consideration the presence of C, H, O, N, S and P and excluding other elements. During formula assignment, peaks were aligned using a 0.5 ppm threshold to enable cross-sample comparisons.

Aligned data reports were processed using the R package ftmsRanalysis[30] to 1) remove peaks that either were outside the desired m/z range (200 m/z – 900 m/z) or had an isotopic signature, 2) calculate a number of derived statistics (Kendrick defect, double-bond equivalent, aromaticity index, nominal oxidation state of carbon, standard Gibb's Free Energy of carbon oxidation), 3) classify chemical formula based upon different boundary sets[31-33], and 4) organize the data into a common framework[34-37]. Because peak intensities cannot be used to infer concentration, all peak intensities were changed to binary presence/absence as recommended by Graham, et al. [38] or Minor, et al. [28] The absence of a peak suggests that it is below the limit of detection. The data is included in the dataset published online under Wells, et al. [39]. The three samples taken at each surface water site per time point as well as the samples taken from the three pushpoints at each time point were used as replicates and averaged per river compartment and time point.

From the 16 compound classes assigned using boundary set 1[31] in the ftmsRanalysis R package, seven compound classes were chosen and grouped in poorly biodegradable compounds with rather complex molecule structures, multiple bonds and/or aromatic rings (condensed hydrocarbon-, lignin-, tannin-, unsaturated hydrocarbon-likes) and in easily biodegradable compounds (carbohydrate-, lipid-, protein-likes).

VFLUX model to calculate seepage flux

In order to calculate seepage flux velocities and directions, the MATLAB based program VFLUX 2.0 was applied. VFLUX 2.0 determines amplitude reduction ratios and phase shifts of daily temperature fluctuations between pairs of temperature sensors by using dynamic harmonic regression[40]. The heat transport model used in this study was introduced by McCallum, et al. [41] and combines amplitude ratios and phase shift information. The first and last 24 hours of the calculated seepage fluxes were discarded, as recommended by Gordon, et al. [40], to remove edge effects that arise from the dynamic harmonic regression.

References

1 Trinh, T. *et al.* Seasonal variations in fate and removal of trace organic chemical contaminants while operating a full-scale membrane bioreactor. *Sci. Total Environ.* **550,** 176-183, doi:https://doi.org/10.1016/j.scitotenv.2015.12.083 (2016).

2 Drewes, J. E. *et al.* Tuning the performance of a natural treatment process using metagenomics for improved trace organic chemical attenuation. *Water Sci Technol* **69,** 628-633, doi:10.2166/wst.2013.750 (2014).

3 Rutere, C., Knoop, K., Posselt, M., Ho, A. & Horn, M. A. Ibuprofen Degradation and Associated Bacterial Communities in Hyporheic Zone Sediments. *Microorganisms* **8,** 1245, doi:10.3390/microorganisms8081245 (2020).

4 Alidina, M., Li, D., Ouf, M. & Drewes, J. E. Role of primary substrate composition and concentration on attenuation of trace organic chemicals in managed aquifer recharge systems. *J Environ Manage* **144,** 58-66, doi:https://doi.org/10.1016/j.jenvman.2014.04.032 (2014).

5 Henning, N., Kunkel, U., Wick, A. & Ternes, T. A. Biotransformation of gabapentin in surface water matrices under different redox conditions and the occurrence of one major TP in the aquatic environment. *Water Res.* **137,** 290-300, doi:10.1016/j.watres.2018.01.027 (2018).

6 Radke, M., Lauwigi, C., Heinkele, G., Mürdter, T. E. & Letzel, M. Fate of the Antibiotic Sulfamethoxazole and Its Two Major Human Metabolites in a Water Sediment Test. *Environ. Sci. Technol.* **43,** 3135-3141, doi:10.1021/es900300u (2009).

7 Nödler, K., Tsakiri, M. & Licha, T. The impact of different proportions of a treated effluent on the biotransformation of selected micro-contaminants in river water microcosms. *Int J Environ Res Public Health* **11,** 10390-10405, doi:10.3390/ijerph111010390 (2014).

8 Jaeger, A. *et al.* Using recirculating flumes and a response surface model to investigate the role of hyporheic exchange and bacterial diversity on micropollutant half-lives. *Environ. Sci.: Process. Impacts* **21,** 2093-2108, doi:10.1039/C9EM00327D (2019).

9 Storck, F. R., Skark, C., Remmler, F. & Brauch, H. J. Environmental fate and behavior of acesulfame in laboratory experiments. *Water Sci Technol* **74,** 2832-2842, doi:10.2166/wst.2016.452 (2016).

10 Dummer, N. M. 4(5)-Methylbenzotriazole: a review of the life-cycle of an emerging contaminant. *Rev. Environ. Sci. Biotechnol.* **13,** 53-61, doi:10.1007/s11157-013-9318-y (2014).

11 Writer, J. H., Antweiler, R. C., Ferrer, I., Ryan, J. N. & Thurman, E. M. In-stream attenuation of neuro-active pharmaceuticals and their metabolites. *Environ. Sci. Technol.* **47,** 9781-9790, doi:10.1021/es402158t (2013).
12 Ghattas, A.-K., Fischer, F., Wick, A. & Ternes, T. A. Anaerobic biodegradation of (emerging) organic contaminants in the aquatic environment. *Water Res.* **116,** 268-295, doi:https://doi.org/10.1016/j.watres.2017.02.001 (2017).
13 Riml, J., Wörman, A., Kunkel, U. & Radke, M. Evaluating the fate of six common pharmaceuticals using a reactive transport model: Insights from a stream tracer test. *Sci Total Environ* **458-460,** 344-354, doi:https://doi.org/10.1016/j.scitotenv.2013.03.077 (2013).
14 Kunkel, U. & Radke, M. Fate of pharmaceuticals in rivers: deriving a benchmark dataset at favorable attenuation conditions. *Water Res.* **46,** 5551-5565, doi:10.1016/j.watres.2012.07.033 (2012).
15 Huntscha, S., Velosa, D., Schroth, M. & Hollender, J. Degradation of Polar Organic Micropollutants during Riverbank Filtration: Complementary Results from Spatiotemporal Sampling and Push-Pull Tests. *Environ. Sci. Technol.* **47**, doi:10.1021/es401802z (2013).
16 Alidina, M., Li, D. & Drewes, J. E. Investigating the role for adaptation of the microbial community to transform trace organic chemicals during managed aquifer recharge. *Water Res.* **56,** 172-180, doi:https://doi.org/10.1016/j.watres.2014.02.046 (2014).
17 Schaper, J. L. *et al.* Fate of trace organic compounds in the hyporheic zone: Influence of retardation, the benthic biolayer, and organic carbon. *Environ. Sci. Technol.* **53,** 4224-4234, doi:10.1021/acs.est.8b06231 (2019).
18 Schmidt, R. & Brockmeyer, R. Occurrence and behavior of expectorants, analgesics, xylometazoline and their metabolites in water and during bank filtration. *Vom Wasser* **98,** 37-54 (2002).
19 Panayiotopoulos, C. *Atlas of Epilepsies*. (London: Springer London, 2010).
20 Herrmann, M., Menz, J., Olsson, O. & Kummerer, K. Identification of phototransformation products of the antiepileptic drug gabapentin: Biodegradability and initial assessment of toxicity. *Water Res.* **85,** 11-21, doi:10.1016/j.watres.2015.08.004 (2015).
21 Zhang, X. *et al.* Removal of acidic pharmaceuticals by small-scale constructed wetlands using different design configurations. *Sci Total Environ* **639,** 640-647, doi:https://doi.org/10.1016/j.scitotenv.2018.05.198 (2018).
22 Schaper, J. L. *et al.* The fate of polar trace organic compounds in the hyporheic zone. *Water Res.* **140,** 158-166, doi:10.1016/j.watres.2018.04.040 (2018).
23 Sui, Q., Huang, J., Deng, S., Chen, W. & Yu, G. Seasonal Variation in the Occurrence and Removal of Pharmaceuticals and Personal Care Products in Different Biological Wastewater Treatment Processes. *Environ. Sci. Technol.* **45,** 3341-3348, doi:10.1021/es200248d (2011).
24 Jaeger, A. *et al.* Spatial and temporal variability in attenuation of polar organic micropollutants in an urban lowland stream. *Environ. Sci. Technol.* **53,** 2383-2395, doi:10.1021/acs.est.8b05488 (2019).
25 Dittmar, T., Koch, B. P., Hertkorn, N. & Kattner, G. A simple and efficient method for the solid-phase extraction of dissolved organic matter (SPE-DOM) from seawater. *Limnol and Oceanogr-Meth* **6,** 230-235 (2008).
26 Tolić, N. *et al.* Formularity: Software for automated formula assignment of natural and Other organic matter from ultrahigh-resolution mass spectra. *Anal. Chem.* **89,** 12659-12665, doi:10.1021/acs.analchem.7b03318 (2017).
27 Kujawinski, E. B. & Behn, M. D. Automated analysis of electrospray ionization fourier transform ion cyclotron resonance mass spectra of natural organic matter. *Anal. Chem.* **78,** 4363–4373 (2006).
28 Minor, E. C., Steinbring, C. J., Longnecker, K. & Kujawinski, E. B. Characterization of dissolved organic matter in Lake Superior and its watershed using ultrahigh resolution mass spectrometry. *Org Geochem* **43,** 1-11 (2012).

29 Tfaily, M. M. *et al.* Sequential extraction protocol for organic matter from soils and sediments using high resolution mass spectrometry. *Anal. Chim. Acta* **972,** 54-61, doi:https://doi.org/10.1016/j.aca.2017.03.031 (2017).
30 ftmsRanalysis: Analysis and visualization tools for FT-MS data. R package version 1.0.0. (2019).
31 Kim, S., Kramer, R. W. & Hatcher, P. G. Graphical method for analysis of ultrahigh-resolution broadband mass spectra of natural organic matter, the Van Krevelen diagram. *Anal. Chem.* **75,** 5336-5344, doi:10.1021/ac034415p (2003).
32 Bailey, V. L., Smith, A. P., Tfaily, M., Fansler, S. J. & Bond-Lamberty, B. Differences in soluble organic carbon chemistry in pore waters sampled from different pore size domains. *Soil Biol Biochem* **107,** 133-143 (2017).
33 Rivas-Ubach, A. *et al.* Moving beyond the van Krevelen diagram: A new stoichiometric approach for compound classification in organisms. *Anal. Chem.* **90,** 6152-6160 (2018).
34 Hughey, C. A., Hendrickson, C. L., Rodgers, R. P., Marshall, A. G. & Qian, K. Kendrick mass defect spectrum: A compact visual analysis for ultrahigh-resolution broadband mass spectra. *Anal. Chem.* **73,** 4676–4681 (2001).
35 Koch, B. P. & Dittmar, T. From mass to structure: an aromaticity index for high-resolution mass data of natural organic matter. *Rapid Commun Mass Sp* **30,** 250-250, doi:10.1002/rcm.7433 (2006).
36 LaRowe, D. E. & Van Cappellen, P. Degradation of natural organic matter: A thermodynamic analysis. *Geochim. Cosmochim. Acta* **75,** 2030–2042 (2011).
37 Tfaily, M. M. *et al.* Advanced solvent based methods for molecular characterization of soil organic matter by high-resolution mass spectrometry. *Anal. Chem.* **87,** 5206-5215, doi:10.1021/acs.analchem.5b00116 (2015).
38 Graham, E. B. *et al.* Carbon Inputs From Riparian Vegetation Limit Oxidation of Physically Bound Organic Carbon Via Biochemical and Thermodynamic Processes. *Journal of Geophysical Research: Biogeosciences* **122,** 3188-3205, doi:10.1002/2017JG003967 (2017).
39 Wells, J. R. *et al.* WHONDRS 48 hour diel cycling study at the Erpe River, Germany. (2019).
40 Gordon, R. P., Lautz, L. K., Briggs, M. A. & McKenzie, J. M. Automated calculation of vertical pore-water flux from field temperature time series using the VFLUX method and computer program. *J Hydrol* **420-421,** 142-158, doi:https://doi.org/10.1016/j.jhydrol.2011.11.053 (2012).
41 McCallum, A. M., Andersen, M. S., Rau, G. C. & Acworth, R. I. A 1-D analytical method for estimating surface water–groundwater interactions and effective thermal diffusivity using temperature time series. *Water Resour. Res.* **48**, doi:doi:10.1029/2012WR012007 (2012).
